# Workplace productivity losses due to multimorbidity: findings from an Australian longitudinal population survey, 2009–21

**DOI:** 10.1093/pubmed/fdaf132

**Published:** 2025-10-10

**Authors:** Mohammad Afshar Ali, Syed Afroz Keramat, Christine Y Lu

**Affiliations:** Sydney Pharmacy School, The University of Sydney, Level 13, Kolling Building, Royal North Shore Hospital, St Leonards, NSW 2065, Australia; Centre for Health Services Research, Faculty of Health, Medicine and Behavioural Sciences, The University of Queensland, Level 5, UQ Health Sciences Building, Fig Tree Lane, Herston, QLD 4006, Australia; Sydney Pharmacy School, The University of Sydney, Level 13, Kolling Building, Royal North Shore Hospital, St Leonards, NSW 2065, Australia; Kolling Institute, Faculty of Medicine and Health, The University of Sydney and the Northern Sydney Local Health District, Level 13, Kolling Building, Royal North Shore Hospital, St Leonards, NSW 2065, Australia

**Keywords:** health services, morbidity and mortality, public health

## Abstract

**Background:**

While productivity loss has been studied in various populations, the impact of multimorbidity on workplace productivity at a population level remains understudied. This study estimates the productivity losses attributable to multimorbidity.

**Method:**

Using data from four waves of the Household, Income and Labour Dynamics in Australia (HILDA) survey, we investigated the relationship between multimorbidity and productivity loss. Negative binomial and logistic regression models were employed to analyze absenteeism, presenteeism, and working hour tension as measures of productivity loss.

**Results:**

We found a significant association between multimorbidity and increased absenteeism, presenteeism and working hour tension. After controlling for socio-economic, demographic, health, and workplace-related factors, individuals with multimorbidity had a 1.07-fold higher rate of absenteeism (incidence rate ratios: 1.07; 95% CI: 1.02–1.13) compared to those without serious illness. Their odds of experiencing presenteeism were three times higher, and the incidence of working hour tension was 32% higher. On average, the annual cost of absenteeism was AU$265.20 higher for individuals with multimorbidity than for those without serious illness.

**Conclusion:**

Our results underscore the need for evidence-based workplace policies to support the productivity and well-being of employees living with multimorbidity.

## Introduction

Multimorbidity is a significant public health challenge and a key driver of health inequalities in many countries.^1^^,^^2^ In the literature, the terms “multimorbidity” and “comorbidity” are often used interchangeably to refer to the coexistence of two or more chronic conditions in an individual.^3^ In Australia, it is estimated that 38% of the population (9.7 million people) had at least two long-term health conditions in 2022, with nearly half of those aged 65 and over being affected.^4^ Due to an aging population, higher survival rates, and the widespread presence of lifestyle-related risk factors (such as smoking, obesity, and physical inactivity), the prevalence of multimorbidity is expected to rise in the coming years.^5^

Evidence from high-income countries shows that, in addition to negative health outcomes, multimorbidity imposes substantial economic burdens on individuals.^6^ Research indicates that patients with multiple comorbidities tend to have higher healthcare utilization, including more outpatient visits, hospitalizations, medical equipment, and medications.^7^ These economic costs, resulting from a higher treatment burden, not only involve significant medical expenses but also include lost income due to unavoidable work absences.^8^^,^^9^ However, there are relatively few studies examining the relationship between multimorbidity and productivity in high-income countries, including Australia.^10^

Existing studies on multimorbidity and productivity loss have focused on various populations. Most research has primarily examined middle-aged and older adults in the general population.^10^^,^^11^ However, there is limited research on the productivity implications of multimorbidity among younger adults.^12^ The existing literature is also diverse in terms of study designs, the number and types of comorbidities, and the outcome measures used.^13^^,^^14^ Notably, many studies have focused on the impact of self-reported ill health or specific conditions, such as arthritis and other musculoskeletal disorders,^15^^,^^16^ asthma,^16^ chronic obstructive pulmonary disease,^16^ obesity,^17^ hypertension,^18^ and mental illness.^19^ Although a few studies conducted in the Australian context have explored the impact of absenteeism on productivity loss,^10^^,^^20^ they frequently employ a narrow definition of absenteeism, conflating it with sick leave.

To our knowledge, no research has been conducted on the association between overall multimorbidity and work productivity at the population level using comprehensive measures such as absenteeism, presenteeism, and working hour tension, based on nationally representative data. Gaining deeper insight into the impact of multimorbidity on work productivity could enhance our understanding of its burden on the employed workforce and help inform targeted policy interventions. Therefore, the aim of this study is to (i) examine the association between multimorbidity and workplace productivity, and (ii) estimate the productivity losses associated with multimorbidity.

## Methods

### Data source

The data for this study were sourced from the Household, Income and Labour Dynamics in Australia (HILDA) Survey. HILDA is a nationally representative, household-based panel survey that has been tracking adults aged 15 and older in participating households annually since 2001. The survey collects data on various aspects of life in Australia, including household composition, income and employment, health and well-being, education and skills, family dynamics, housing, material deprivation, and life events. Data collection involved interviews and a self-completion questionnaire, conducted in accordance with the University of Melbourne’s ethical guidelines to ensure confidentiality and uphold ethical research standards.

The HILDA Survey, initiated in 2001, employs a multi-stage sampling method to create a representative sample of Australian households. It’s a longitudinal study, tracking the same individuals and households annually. The first wave included over 13 000 adults from 7000+ households (66% response rate), with new participants added through household changes and booster samples since 2011.^21^^,^^22^ HILDA boasts a high retention rate of ~96%, exceeding international benchmarks.^21^ Further details on the survey design, data collection procedures, and participant recruitment are available in other sources.^21^

### Analytic sample and missing data

**Figure 1:**
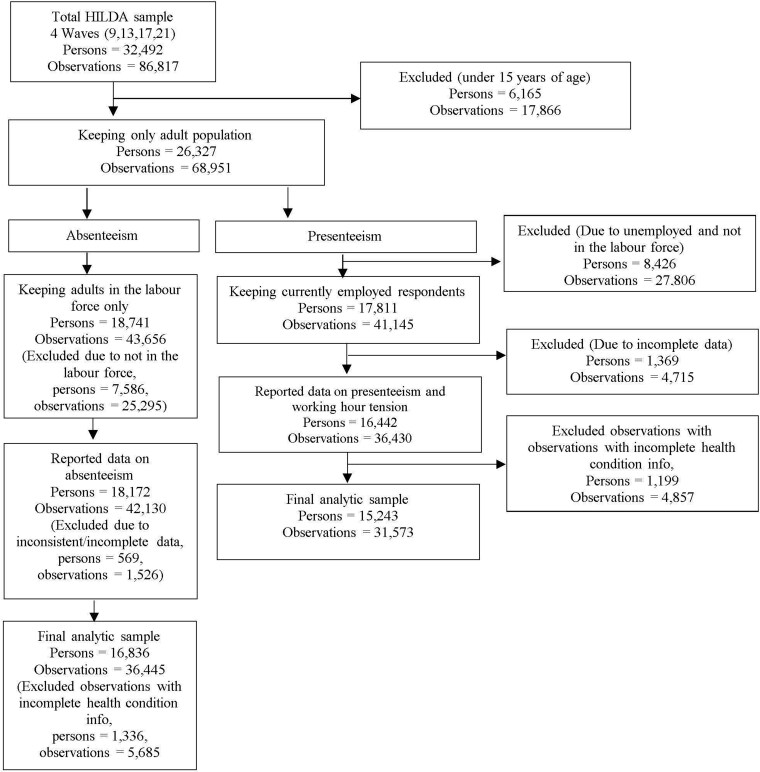
Participants flow into the analytic sample, and missing data.

We utilized data from Waves 9, 13, 17, and 21 of the HILDA Survey, corresponding to the years 2009, 2013, 2017, and 2021, to estimate productivity loss related to multimorbidity. These waves were specifically chosen because they included the comprehensive health survey module, which has been administered every four years since Wave 9. Given the study’s focus on the productivity costs of multimorbidity, the sample was restricted to respondents aged 15 years and over. For the absenteeism analysis, we included individuals in the labor force (defined as those either employed or unemployed but actively seeking work). For the analyses of presenteeism and working hour tension, only employed respondents aged 15 years and over were included. Adult household members were eligible if they had responded to the question on serious illness. To reduce potential biases, we further restricted the analysis to individuals with complete data for absenteeism, presenteeism, and working hour tension. Based on these inclusion and exclusion criteria, we constructed unbalanced panel datasets. The unbalanced pooled dataset for absenteeism included 36 445 person-year observations from 16 836 unique individuals. The final analytic sample for presenteeism and working hour tension comprised 31 573 person-year observations from 15 243 unique individuals. The flow of the final analytic samples and handling of missing data are outlined in Fig. 1.

As this was a multi-wave longitudinal study, we included all individuals who provided data on productivity outcomes (namely, absence days, presenteeism, and working hour tension). In line with the HILDA survey design, respondents were classified as employed or unemployed if they reported being in either status at any point during the preceding 12 months. Employment-related characteristics (e.g. contract type, firm size, occupation group, supervisory responsibility, union membership, and job satisfaction) were drawn from the same wave in which employment status was defined. For waves where these details were missing, we applied a last observation carried forward approach to impute missing values, as supported by previous research, thereby maximizing sample retention based on available outcome data.

### Measures

#### Dependent variables

To evaluate the productivity loss associated with multimorbidity, we used three outcomes: absenteeism, presenteeism, and working hour tension.^23^ The primary outcome, absenteeism, was measured by asking participants to report the number of days they had missed work in the past 12 months. Total leave was calculated by adding up the days of paid annual leave, sick leave, unpaid leave, and other leave types (such as maternity, paternity, and bereavement leave) taken during the previous 12 months. Recognizing that unemployment can be considered a form of involuntary absence, we also included the number of days of unemployment in the past fiscal year as part of our total absence measure. Thus, total absence was the sum of days taken for paid annual leave, sick leave, unpaid leave, other leave, and unemployment in the past 12 months.

Presenteeism was assessed using the Short-Form 36 Health Survey (SF-36), administered annually as part of the HILDA Survey. The SF-36 includes questions related to both physical and emotional role limitations, specifically asking participants if they had experienced any of the following in the past four weeks due to physical or emotional problems: cutting down the amount of time spent on work or other activities, accomplishing less than they would like, or not doing work or other activities as carefully as usual. Each of these six questions used a binary response format (yes/no). An individual was considered to have experienced presenteeism in the previous four weeks if they responded “yes” to any of these six questions.

Working hour tension, a count variable, was calculated by subtracting the usual weekly hours worked (across all jobs) from the preferred weekly hours worked, following prior research.^23^^,^^24^ This measure captures the discrepancy between actual and desired work hours. If actual work hours were greater than or equal to preferred hours, the resulting tension was zero. Otherwise, working hour tension was a non-negative integer representing the difference between preferred and actual work hours.

### Key exposure variable

The key exposure variable in this study, capturing the presence of chronic conditions, was determined based on self-reported participant data. Using the HILDA Survey, respondents were asked whether they had ever been diagnosed with any of 11 specified serious illnesses or medical conditions by a medical practitioner. These conditions included hypertension, heart disease, type 1 diabetes, type 2 diabetes, chronic bronchitis/emphysema, cancer, asthma, arthritis/osteoporosis, anxiety/depression, other mental health conditions, and circulatory disease. Based on their responses, study participants were grouped into three cohorts: (i) those with no reported chronic conditions, (ii) those with a single chronic condition, and (iii) those with two or more co-occurring chronic conditions (multimorbidity).^25^^,^^26^

### Confounders

This analysis accounted for several potential confounding variables linked to productivity loss, as identified in previous studies.^23^^,^^27^ By adjusting for these confounders, we aimed to ensure that the observed relationship between multimorbidity and productivity loss was not attributable to other factors. The confounders included age (15–24, 25–39, 40–64, and 65+ years), gender (male and female), relationship status (partnered and unpartnered), annual household disposable income quintile (quintile 1 [poorest] to quintile 5 [richest]), Indigenous status (non-Indigenous versus Indigenous), region of residence (major cities versus rural and remote areas), BMI (underweight, healthy weight, overweight, and obese), disability status (no versus yes), smoking (non-smoker and current smoker), alcohol consumption (non-drinker and current drinker), physical activity (below recommended versus recommended level), firm size (small, medium, and large), employment contract type (casual, fixed-term, and permanent), employment status (full-time versus part-time), occupation (managers, professionals, technicians and trade workers, community and personal service workers, clerical and administrative workers, sales workers, machinery operators and drivers, and laborers), supervisory responsibility (no versus yes), union membership (no versus yes), paid holiday leave (no versus yes), sick leave (no versus yes), and overall job satisfaction (rated on a scale from 0 [totally dissatisfied] to 10 [totally satisfied]). As part of a sensitivity analysis, hospital admission in the past 12 months was included as a covariate to serve as a proxy for disease severity, given that HILDA Survey does not capture direct severity measures. However, we acknowledge that hospital admission is closely correlated with multimorbidity and may therefore reflect overlapping rather than independent risk. Further information on the construction of these confounders is provided in Table A1.

### Statistical analyses

In the first stage of the analysis, we presented descriptive statistics for the study participants. Means and standard deviations were calculated for continuous variables, while frequencies and percentages were used for categorical variables. In the second stage, we examined bivariate relationships between the key outcome variables and the primary exposure of interest—multimorbidity. We reported the mean absenteeism, the average proportion of presenteeism among participants, and mean working hour tension, stratified by multimorbidity status (i.e. individuals without serious illness, with one serious illness, and with multimorbidity). Independent sample *t*-test were used to examine the statistical significance of mean differences in absenteeism and working hour tension across groups. For presenteeism, a dichotomous variable, we used Pearson’s chi-square (χ2) test to examine differences between the three groups. We applied negative binomial regression to investigate the impact of multimorbidity on absenteeism and working hour tension. The negative binomial regression model was selected due to the over-dispersion and the high frequency of zero counts in the absenteeism data. The distribution of both outcome variables is illustrated in Fig. A1, which demonstrates a strong right skew and clustering near zero. By addressing excess zeros and overdispersion, negative binomial regression provides more accurate and reliable parameter estimates than standard regression models. To assess the relationship between multimorbidity and presenteeism, we used logistic regression, appropriate for the binary nature of the outcome. Results from the negative binomial regression models were reported as adjusted incidence rate ratios (aIRR), and results from the logistic regression were reported as adjusted odds ratios (aOR), both with 95% confidence intervals (CI). Statistical significance was determined using a threshold of *P*-value < .05. We assessed multicollinearity among covariates using the Variance Inflation Factor (VIF) test. All statistical analyses were conducted using R version 4.3.1 (R Foundation for Statistical Computing, Vienna, Austria).

### Estimates of multimorbidity-attributable costs of absenteeism

We calculated the average annual costs of absenteeism associated with multimorbidity among Australian adults currently in the labor force. To estimate the total absenteeism costs, we applied the following formula:


\begin{eqnarray*} && Costs\ of\ absent eeism =\\&&\quad \left( Additional\ absent\ days\ in\ the\ past\ 12\ months\right)\ \\&&\quad x\ \left( Average\ daily\ gross\ wages\ and\ salary\right). \end{eqnarray*}

We estimated the total number of leave days taken in the past 12 months using two complementary approaches. First, we calculated the mean difference in leave days between individuals with multimorbidity and those without serious illness (see Table A3). Second, we estimated the average marginal (partial) effects of multimorbidity, assuming no random effects, to further quantify its impact. To calculate average daily gross wages and salaries, we used two data sources (see Table A4). We first derived mean wages and salaries from our HILDA study sample. To enhance the robustness of our estimates, we also incorporated data on gross wages and salaries from the Australian Bureau of Statistics. Specifically, HILDA wage data were used for the individual-level analysis, while ABS data were employed to validate and supplement our findings at the aggregate level.

## Results

### Descriptive statistics

Table 1 presents the distribution of the 36 445 observations included in this study. Of these, 76.80% (*n* = 27 997) reported no serious illness, 13.50% (*n* = 4916) reported only one serious health condition, and 9.70% (*n* = 3532) were living with two or more chronic conditions. The table also outlines the socio-demographic, health, and employment characteristics of the full analytic sample—including both employed and unemployed individuals—stratified by cohort. Table A2 presents similar information, but is restricted to the subset of employed individuals, also stratified by cohort. A detailed age-specific and overall population-level breakdown of individual chronic conditions among those with multimorbidity is provided in Table A5.

**Table 1:** Distribution of the analytic sample (sociodemographic, health, and job-related characteristics) by cohort (full sample).

Variable	No serious illness (*n* = 27 997)		One serious illness (*n* = 4916)		Two or more serious illness (*n* = 3532)		All cohort (*n* = 36 445)	
	*n*	%	*n*	%	*n*	%	*n*	%
**Age**								
15–24 years	5988	21.39	578	11.76	508	14.38	7074	19.41
25–39 years	10 533	37.62	1364	27.75	918	25.99	12 815	35.16
40–64 years	11 031	39.40	2638	53.66	1754	49.66	15 423	42.32
65 years and above	445	1.59	336	6.83	352	9.97	1133	3.11
**Gender**								
Male	14 892	53.19	2403	48.88	1488	42.13	18 783	51.54
Female	13 105	46.81	2513	51.12	2044	57.87	17 662	48.46
**Relationship status**								
Partnered	17 269	61.68	3168	64.44	1976	55.95	22 413	61.50
Unpartnered	10 728	38.32	1748	35.56	1556	44.05	14 032	38.50
**Highest education level**								
Year 12 and below	9976	35.63	1538	31.29	1213	34.34	12 727	34.92
Professional qualifications	10 795	38.56	2138	43.49	1613	45.67	14 546	39.91
University qualifications	7226	25.81	1240	25.22	706	19.99	9172	25.17
**Household yearly disposable income**								
Q1 (poorest)	5486	19.64	848	17.28	880	24.98	7214	19.84
Q2	5687	20.36	890	18.14	712	20.21	7289	20.04
Q3	5765	20.63	899	18.32	625	17.74	7289	20.04
Q4	5579	19.97	1075	21.91	635	18.02	7289	20.04
Q5 (richest)	5422	19.41	1195	24.35	671	19.05	7288	20.04
**Indigenous status**								
Not of Indigenous origin	27 954	99.85	4909	99.86	3525	99.80	36 388	99.84
Indigenous origin	43	0.15	7	0.14	7	0.20	57	0.16
**Region of residence**								
Major city	19 584	69.95	3313	67.39	2356	66.70	25 253	69.29
Regional city and remote area	8413	30.05	1603	32.61	1176	33.30	11 192	30.71
**BMI**								
Underweight	689	2.46	75	1.53	47	1.33	811	2.23
Healthy weight	12 314	43.98	1595	32.45	967	27.38	14 876	40.82
Overweight	9737	34.78	1799	36.59	1121	31.74	12 657	34.73
Obesity	5257	18.78	1447	29.43	1397	39.55	8101	22.23
**Long-term condition or disability**								
Yes	2563	9.15	1339	27.24	1812	51.30	30 731	84.32
No	25 434	90.85	3577	72.76	1720	48.70	5714	15.68
**Smoking status**								
Non-smoker	23 042	82.30	4168	84.78	2761	78.17	29 971	82.24
Current smoker	4955	17.70	748	15.22	771	21.83	6474	17.76
**Alcohol consumption**								
Current drinker	3831	13.68	677	13.77	560	15.86	31 377	86.09
Non-drinker	24 166	86.32	4239	86.23	2972	84.14	5068	13.91
**Physical activity**								
Less than the recommended level	17 502	62.51	3218	65.46	2557	72.40	23 277	63.87
Recommended level	10 495	37.49	1698	34.54	975	27.60	13 168	36.13
**Firm size**								
Small (1–19 employees)	12 116	43.28	2095	42.62	1669	47.25	15 880	43.57
Medium (20–99 employees)	7672	27.40	1320	26.85	886	25.08	9878	27.10
Large (≥100 employees)	8209	29.32	1501	30.53	977	27.66	10 687	29.32
**Employment contract**								
Casual	6128	21.89	1006	20.46	892	25.25	3478	9.54
Fixed term	2726	9.74	441	8.97	311	8.81	24 941	68.43
Permanent	19 143	68.38	3469	70.57	2329	65.94	8026	22.02
**Employment nature**								
Fulltime	18 620	66.51	3167	64.42	1916	54.25	23 703	65.04
Parttime	8592	30.69	1634	33.24	1431	40.52	11 657	31.99
Unemployed	785	2.80	115	2.34	185	5.24	1085	2.98
**Occupation**								
Managers	3822	13.65	742	15.09	441	12.49	9119	25.02
Professionals	7047	25.17	1268	25.79	804	22.76	5005	13.73
Technicians and trades workers	3891	13.90	604	12.29	385	10.90	4880	13.39
Community and personal service workers	3126	11.17	572	11.64	533	15.09	4231	11.61
Clerical and administrative workers	3604	12.87	659	13.41	472	13.36	4735	12.99
Sales workers	2480	8.86	358	7.28	303	8.58	3141	8.62
Machinery operators and drivers	1604	5.73	316	6.43	238	6.74	2158	5.92
Laborers	2423	8.65	397	8.08	356	10.08	3176	8.71
**Supervisory responsibilities**								
No	15 408	55.03	2825	57.47	2182	61.78	20 415	56.02
Yes	12 589	44.97	2091	42.53	1350	38.22	16 030	43.98
**Union membership**								
No	22 122	79.02	3805	77.40	2699	76.42	28 626	78.55
Yes	5875	20.98	1111	22.60	833	23.58	7819	21.45
**Paid holiday or sick leave**								
No	6910	24.68	1133	23.05	974	27.58	8886	24.38
Yes	21 193	75.70	3802	77.34	2564	72.59	27 428	75.26
**Hospital admission**								
No	26 068	93.11	4427	90.05	2912	82.45	33 407	91.66
Yes	1929	6.89	489	9.95	620	17.55	3038	8.34
**Functional disability**								
No	26 746	95.53	4301	87.49	2447	69.28	33 494	91.90
Yes	1251	4.47	615	12.51	1085	30.72	2951	8.10
**Overall job satisfaction, mean (SD)**	7.73 (1.53)		7.79 (1.56)		7.68 (1.70)		7.73 (1.55)	

Among individuals in the multimorbidity cohort, the majority were middle-aged (49.66%), partnered (55.95%), non-Indigenous (99.80%), and residing in major cities (66.70%). Additionally, 39.24% of this cohort were classified as obese, 51.27% reported having a long-term health disability, 21.72% were current smokers, 84.40% consumed alcohol, and 72.57% did not meet the recommended level of physical activity (Table 1). Regarding employment characteristics, a substantial proportion of individuals with multimorbidity were employed in small firms (47.25%), held permanent contracts (65.94%), worked in professional occupations (22.76%), had no supervisory responsibilities (61.78%), were not union members (76.42%), and received employment benefits such as paid holiday or sick leave (72.59%). The average job satisfaction score for individuals with multimorbidity was 7.68, with a standard deviation of 1.70 (based on pooled data). Table A2 presents similar socio-demographic and employment characteristics, but focuses exclusively on employed individuals, excluding those who were unemployed.

Table 2 presents the distribution of absenteeism, presenteeism, and working hour tension by multimorbidity status (i.e. individuals without serious illness, with one serious illness, and with multimorbidity). For absenteeism, individuals without serious illness reported an average of 16.8 days per year (SD: 21.6), while those with a single chronic condition and those with multimorbidity reported higher averages of 18.8 days (SD: 24.5) and 17.7 days (SD: 24.5), respectively. Regarding presenteeism, 25.52% of individuals without serious illness reported experiencing it, compared to 37.36% among those with one chronic condition and 61.42% among those with multimorbidity. The mean working hour tension was 1.61 hours (SD: 4.86) among individuals without serious illness, and 1.43 hours (SD: 4.69) among those with one chronic condition. In contrast, individuals with multimorbidity had significantly higher working hour tension, averaging 2.43 hours (SD: 6.15). The *t*-test and χ2-test indicate statistically significant differences between individuals without serious illness and those with multimorbidity (*P*-value < .05) for all three outcomes.

**Table 2:** Outcome variables and multimorbidity status.

Outcome variable	No serious illness		One condition		At least two Conditions		*P*-value
	*N*	%	*N*	%	*N*	%	
**Absence in past 12 months (days)**							
Mean	27 997	16.80	4916	18.80	3532	17.70	<.001
SD		21.60		24.50		24.50	
Min		0		0		0	
Max		336		285		312	
**Presenteeism**							
No	17 902	74.48	2803	62.64	1181	38.58	<.001
Yes	6135	25.52	1672	37.36	1880	61.42	
**Working hour tension (hours)**							
Mean	24 037	1.61	4475	1.43	3061	2.43	<.001
SD		4.86		4.69		6.15	
Min		0		0		0	
Max		65		56		55	

Note: *P*-values are from *t*-tests (absence days, working hour tension) and χ^2^-tests (presenteeism), indicating the probability of differences across the three cohorts.

### Regression modeling

Table 3 presents the results of the adjusted regression analyses, based on 36 445 and 31 573 person-years of observation for absenteeism, presenteeism, and working hour tension, respectively. After adjusting for sociodemographic, health, and job-related factors, individuals with multimorbidity exhibited a 7% higher rate of absenteeism compared to those without serious illness (IRR: 1.07; 95% CI: 1.02–1.13). For context, individuals with a single chronic condition had a modest 4% higher rate of absenteeism (IRR: 1.04; 95% CI: 1.00–1.09). A significant association was also observed between multimorbidity and presenteeism. Individuals with multimorbidity had more than three times the odds of reporting presenteeism than those without serious illness (aOR: 3.11; 95% CI: 2.85–3.40). Those with one chronic condition also had elevated odds of presenteeism, though to a lesser extent (aOR: 1.50; 95% CI: 1.39–1.61). Similarly, individuals with multimorbidity experienced a 34% higher incidence of working hour tension compared to those without serious illness (IRR: 1.34; 95% CI: 1.12–1.61), whereas individuals with a single chronic condition did not show a statistically significant increase (IRR: 0.96; 95% CI: 0.83–1.12).

**Table 3:** Results from the negative binomial regression (models 1 and 3) and logistic regression (model 2).

Variables	Model 1: absenteeism adjusted IRR (95%CI)	Model 2: presenteeism adjusted OR (95%CI)	Model 3: working hour tension adjusted IRR (95%CI)
Intercept	14.65^a^ [12.96–16.55]	0.91 [0.73–1.15]	3.27^a^ [2.11–5.07]
**Chronic illness**			
No serious illness (ref)			
One serious illness	1.04^a^ [1.00–1.09]	1.50^a^ [1.39–1.61]	0.96 [0.83–1.12]
At least two serious illness	1.07^a^ [1.02–1.13]	3.11^a^ [2.85–3.40]	1.34^a^ [1.12–1.61]
**Age**			
15–24 years (ref)			
25–39 years	1.40^a^ [1.34–1.47]	0.99 [0.92–1.09]	0.69^a^ [0.58–0.81]
40–64 years	1.28^a^ [1.22–1.34]	0.82^a^ [0.75–0.89]	0.57^a^ [0.49–0.67]
65 and above	0.86^a^ [0.78–0.94]	0.89 [0.76–1.04]	0.37^a^ [0.27–0.50]
**Gender**			
Male (ref)			
Female	1.16^a^ [1.12–1.19]	1.48^a^ [1.39–1.56]	1.02 [0.91–1.14]
**Relationship status**			
Partnered (ref)			
Unpartnered	0.89^a^ [0.87–0.92]	1.26^a^ [1.19–1.34]	1.11 [0.99–1.13]
**Highest education level**			
Year 12 and below (ref)			
Professional qualifications	1.11^a^ [1.07–1.15]	1.08^a^ [1.01–1.15]	0.99 [0.88–1.13]
University qualifications	1.09^a^ [1.05–1.15]	1.14^a^ [1.05–1.24]	0.98 [0.84–1.14]
**Household yearly disposable income**			
Q1 (poorest) (ref)			
Q2	1.09^a^ [1.05–1.14]	0.90^a^ [0.83–0.97]	0.81^a^ [0.70–0.95]
Q3	1.13^a^ [1.08–1.19]	0.86^a^ [0.79–0.95]	0.63^a^ [0.54–0.74]
Q4	1.17^a^ [1.11–1.22]	0.87^a^ [0.80–0.94]	0.56^a^ [0.47–0.66]
Q5 (richest)	1.14^a^ [1.09–1.20]	0.84^a^ [0.77–0.92]	0.50^a^ [0.43–0.59]
**Indigenous status**			
Not of Indigenous origin (ref)			
Indigenous origin	1.21 [0.84–1.71]	1.08 [0.53–2.24]	1.03 [0.25–4.21]
**Region of residence**			
Major city (ref)			
Regional city and remote area	1.02 [0.98–1.05]	0.94 [0.89–1.00]	0.99 [0.89–1.11]
**BMI**			
Healthy weight (ref)			
Obesity	1.07^a^ [1.03–1.11]	1.11^a^ [1.04–1.19]	1.11 [0.97–1.27]
Overweight	1.06^a^ [1.02–1.09]	1.01 [0.95–1.07]	1.01 [0.90–1.14]
Underweight	0.84^a^ [0.76–0.93]	1.01 [0.91–1.29]	0.98 [0.70–1.39]
**Long-term condition or disability**			
No (ref)			
Yes	1.14^a^ [1.09–1.19]	3.03^a^ [2.80–3.28]	1.22^a^ [1.06–1.41]
**Smoking status**			
Non-smoker (ref)			
Current smoker	0.94^a^ [0.91–0.98]	1.21^a^ [1.12–1.29]	1.27^a^ [1.11–1.45]
**Alcohol consumption**			
Non-drinker (ref)			
Current drinker	1.15^a^ [1.11–1.20]	0.97 [0.90–1.05]	0.91 [0.79–1.05]
**Physical activity**			
Less than the recommended level (ref)			
Recommended level	0.99 [0.96–1.02]	0.76^a^ [0.72–0.80]	1.02^a^ [0.92–1.13]
**Firm size**			
Small (ref)			
Large	1.38^a^ [1.33–1.43]	0.92^a^ [0.86–0.98]	0.63 [0.56–0.72]
Medium	1.31^a^ [1.26–1.35]	0.94 [0.89–1.01]	0.70 [0.62–0.79]
**Employment contract**			
Permanent			
Casual	0.70^a^ [0.65–0.76]	1.05 [0.91–1.20]	1.54^a^ [1.19–1.98]
Fixed term	0.88^a^ [0.83–0.92]	1.02 [0.93–1.11]	0.98 [0.82–1.16]
**Occupation**			
Professionals (ref)			
Managers	0.81^a^ [0.77–0.85]	0.99 [0.90–1.08]	0.68^a^ [0.57–0.81]
Technicians and trades workers	0.89^a^ [0.85–0.95]	0.79^a^ [0.71–0.87]	1.59^a^ [1.32–1.91]
Community and personal service workers	0.90^a^ [0.86–0.95]	0.87^a^ [0.79–0.96]	2.21^a^ [1.84–2.66]
Clerical and administrative workers	0.92^a^ [0.88–0.98]	0.79^a^ [0.72–0.87]	1.15 [0.96–1.37]
Sales workers	0.82^a^ [0.77–0.87]	0.89 [0.80–1.01]	1.70^a^ [1.38–2.11]
Machinery operators and drivers	0.82^a^ [0.77–0.88]	0.71^a^ [0.62–0.81]	1.86^a^ [1.46–2.38]
Laborers	0.77^a^ [0.72–0.82]	0.82^a^ [0.73–0.92]	2.33^a^ [1.88–2.90]
**Supervisory responsibilities**			
Yes (ref)			
No	0.86^a^ [0.84–0.89]	1.08^a^ [1.03–1.15]	1.56^a^ [1.41–1.74]
**Union membership**			
Yes (ref)			
No	0.72^a^ [0.70–0.75]	1.02 [0.96–1.09]	1.02 [0.90–1.16]
**Paid holiday or sick leave**			
Yes (ref)			
No	0.52^a^ [0.49–0.56]	0.95 [0.83–1.08]	1.48^a^ [1.16–1.89]
**Overall job satisfaction**	0.97^a^ [0.96–0.98]	0.83^a^ [0.81–0.84]	0.89^a^ [0.86–0.92]
N	Persons = 16 836 Observations = 36 445	Persons = 15 243 Observations = 31 573	Persons = 15 243 Observations = 31 573
Mean VIF (Max)	1.60 (4.53)	1.63 (4.85)	1.62 (4.81)

Notes: 95% CIs are reported in parentheses; ^a^Indicate significance at the 5% level; Abbreviations: IRR, incidence rate ratio; AOR, adjusted odds ratio; Ref, reference category; VIF, Variance Inflation Factor.

As part of model diagnostics, we assessed multicollinearity using VIF statistics. In Model 1, the mean (maximum) VIF was 1.60 (4.53), indicating no multicollinearity concerns. Similarly, Models showed a mean (maximum) VIF of 1.63 (4.85), and Model 3 reported 1.62 (4.81), confirming the absence of significant multicollinearity across all models.

We explored whether the severity of chronic conditions moderated the relationship between multimorbidity and workplace productivity loss. As the HILDA survey does not capture direct measures of disease severity, we used hospital admission in the past 12 months as a proxy. In the fully adjusted models, hospital admission was significantly associated with absenteeism, presenteeism, and working hour tension. However, its inclusion attenuated the effect of multimorbidity on absenteeism, likely because the risk of hospitalization is itself closely linked to multimorbidity. To test this, we ran an additional regression model (Tables A6 and A7) and confirmed that multimorbidity was significantly associated with hospitalization (aOR: 2.04; 95% CI: 1.79–2.33). We also assessed whether functional disability moderated the effect of multimorbidity by including an interaction term in the same adjusted models. This interaction was not statistically significant, and the results are therefore presented in the supplementary material (Table A7).

### Attributable costs of absenteeism due to multimorbidity

Table 4 presents the estimated costs of work absenteeism attributable to multimorbidity. On average, individuals with multimorbidity incurred AU$265.20 more in annual absenteeism costs compared to those without serious illness. Additional details on excess absent days among individuals with multimorbidity and gross wage estimates are provided in Tables A3 and A4, respectively. A stratified analysis by age group revealed that the highest absenteeism-related costs were observed among individuals aged 40–64 and 25–39, with annual wage losses of AU$306.00 and AU$297.60 per person, respectively.

**Table 4:** Costs of absenteeism attributed to multimorbidity.

Types of productivity costs	Unit wages estimated from HILDA	Unit wages according to ABS
**Mean comparison, unadjusted**		
Person with two or more chronic conditions (whole cohort)	198.90	247.79
Aged 15–24 years	102.60	N/A
Aged 25–39 years	223.20	N/A
Aged 40–64 years	229.50	N/A
Aged 65 years and above	130.50	N/A
**Negative binomial regression effects, adjusted**		
Person with two or more chronic conditions (whole cohort)	265.20	330.38
Aged 15–24 years	136.80	N/A
Aged 25–39 years	297.60	N/A
Aged 40–64 years	306.00	N/A
Aged 65 years and above	174.00	N/A

## Discussion

### Main finding of this study

Individuals with two or more chronic conditions face substantial challenges in modern workplaces, often leading to considerable productivity losses. These conditions not only affect their health but also hinder their ability to perform job tasks efficiently. The relationship between multimorbidity and productivity is complex, involving factors such as absenteeism, presenteeism, and reduced work capacity. This study examines the indirect costs of multimorbidity, focusing on three key measures of productivity: absenteeism, presenteeism, and working hour tension. Using data from four waves of the HILDA survey (2009–21), we employed negative binomial regression and logistic regression to assess how multimorbidity impacts workplace productivity among working-age Australians. Our results show a strong association between multimorbidity and increased absenteeism, highlighting its significant effect on workplace productivity. Specifically, individuals with two or more chronic conditions were absent an average of 1.20 additional days per year compared to those without serious illness. This result aligns with previous research, which found that individuals with high psychological distress had 0.36 more days of absence annually than those with no or low distress.^28^ Furthermore, we found that multimorbidity was associated with a higher likelihood of presenteeism and increased working hour tension, further highlighting its burden on the labor force.

### What is already known on this topic

Existing research on multimorbidity and productivity loss has largely focused on middle-aged and older adults,^10^^,^^11^ with limited studies examining younger populations.^12^ The literature varies in study design, comorbidity types, and outcome measures.^13^^,^^14^ Many studies assess the impact of self-reported ill health or specific conditions like musculoskeletal disorders, asthma, chronic obstructive pulmonary disease, obesity, hypertension, and mental illness.^15^^,^^17–19^ In Australia, research on absenteeism-related productivity loss exists but often uses a narrow definition, equating absenteeism with sick leave.^10^^,^^20^

### What this study adds

While previous research on productivity loss due to multimorbidity has primarily focused on specific chronic conditions or subsets of the population, there is limited understanding of how overall multimorbidity impacts work capacity and labor productivity among working adults at the population level in Australia. This study addresses this gap by examining critical aspects of productivity—absenteeism, presenteeism, and working hour tension—to assess the impact of multimorbidity. This study contributes to the limited research on the relationship between multimorbidity and workplace productivity by utilizing nationally representative longitudinal data.

Our findings also suggest that individuals with multimorbidity are more likely to miss work, consistent with evidence from other developed countries.^10^^,^^12^^,^^20^^,^^26^ We estimate that absenteeism costs related to multimorbidity amount to approximately AU$265.20 per person annually. This estimate aligns closely with findings from a U.S.-based study, which reported an incremental wage loss of US$279.92 per year due to absenteeism among individuals with multimorbidity compared to those without.^29^ Furthermore, our analysis highlights notable age-related differences in wage losses. The highest productivity losses were observed among individuals in the prime working age working groups (25–39 and 40–64 years), compared to younger respondents aged 15–24 years. This likely reflects the general trend of increasing wages with age, experience, and organizational seniority, amplifying the financial impact of health-related work impairments in these groups. This finding aligns with previous research indicating that age is a crucial factor in determining the magnitude of productivity losses, particularly among working-age adults who may be forced to prematurely cease employment due to health issues.^30^ Notably, several Australian studies have shown a strong association between the number of serious health conditions and earlier retirement, further corroborating our findings.^10^^,^^20^^,^^31^

In our study, we found that individuals with multimorbidity experience higher rates of presenteeism. Presenteeism, which refers to working while ill, often arises from health issues, such as functional limitations, that ultimately hinder productivity, efficiency, and accuracy in the workplace. These findings are consistent with previous research. For instance, one study reported higher rates of presenteeism among employees with chronic health conditions compared to their healthier counterparts.^32^ Additionally, another study found that individuals with two or more chronic conditions were 2.3 times more likely to experience presenteeism compared to those without any chronic conditions.^26^

Treatments for chronic diseases, such as medication, surgery, and therapy, often come with debilitating side effects, including reduced physical capacity, cognitive impairment, and emotional distress.^33^ These side effects significantly impair an individual’s ability to concentrate and perform their job responsibilities, thus increasing the likelihood of presenteeism. Moreover, the emotional burden associated with a chronic disease diagnosis and its treatment compounds these challenges.^24^ Employees, driven by financial pressures, fear of job loss, or a sense of responsibility to their colleagues and employer, may feel compelled to continue working despite their illness. This reluctance to take necessary leave can hinder recovery, as adequate rest and appropriate self-care are essential for managing the side effects of treatment.

Our study found that absenteeism decreases with age, with younger adults (25–39) having a higher likelihood of absenteeism than those aged 65 and above. This trend may be due to younger individuals being more likely to work full-time, face workplace stress, and manage caregiving responsibilities, all contributing to increased absenteeism. In contrast, older workers often hold less demanding roles or have greater job flexibility, reducing absenteeism. Additionally, differences in comorbidity prevalence may explain these patterns. Younger adults had higher rates of mental health conditions such as depression and anxiety, which can lead to episodic absences. Meanwhile, older adults were more likely to have chronic conditions like hypertension, diabetes, and arthritis, but they may have better management strategies or workplace accommodations. Furthermore, many older individuals may have left the workforce, further contributing to lower absenteeism rates. Our study also found that females are more likely to be absent from work compared to males, aligning with previous research on productivity loss.^20^^,^^23^

Our analysis also revealed that the absence of paid holiday or sick leave was significantly associated with lower rates of absenteeism compared to those with such entitlements. This suggests that individuals without access to paid leave may be less likely to take time off work when unwell, potentially due to financial constraints or concerns about job insecurity. This finding is particularly noteworthy, as it highlights a structural workplace factor that strongly influences absenteeism behavior. Importantly, even after adjusting for the substantial effect of paid leave access, we continued to observe a significant association between multimorbidity and increased absenteeism. This underscores the independent contribution of multimorbidity to productivity losses, irrespective of employment entitlements. Taken together, these results emphasize the importance of considering both health status and employment protections when developing policies and interventions aimed at reducing productivity costs associated with chronic conditions.

The findings of this study have profound implications for the development and implementation of public policies that enhance workplace outcomes for individuals living with multimorbidity. The substantial indirect costs associated with multimorbidity, particularly the significant impact on workplace productivity, underscore the urgent need for policymakers to address this critical issue. Policies are necessary to encourage companies and relevant stakeholders to adopt flexible work management plans for employees with multimorbidity who require time off for treatment and therapies.^20^^,^^34^ This approach would help minimize unnecessary forced unemployment. Additionally, current healthcare is primarily focused on single-disease-specific care, rather than patient-centered care that considers multimorbidity. As a result, clinical care becomes more complex for individuals with multimorbidity. Therefore, updating clinical guidelines to prioritize patient-centered care and multimorbidity, rather than maintaining a single-disease focus, is essential.

### Limitations and future research direction

Our study has several limitations that should be acknowledged. First, we examined whether the severity of chronic conditions moderated the association between multimorbidity and workplace productivity loss. Because HILDA does not capture direct measures of disease severity, we used hospital admission in the past 12 months as a proxy. In the adjusted models, hospital admission was significantly associated with all productivity outcomes. However, its inclusion attenuated the effect of multimorbidity on absenteeism, likely due to the strong association between multimorbidity and hospitalization. Thus, although hospital admission is associated with productivity outcomes, it does not serve as an independent proxy for disease severity but instead reflects the same underlying risk captured by multimorbidity. Given that previous studies have consistently shown a direct relationship between comorbidity severity and productivity loss,^14^ further research is needed to disentangle the independent effects of multimorbidity and hospitalization. Second, we did not evaluate productivity loss from the employer’s perspective, meaning that indirect costs such as those associated with hiring and training replacement workers were not considered. Incorporating the employer’s perspective with population-level data could offer valuable insights for future research, particularly for events that have minimal impact on overall employment.^35^ Third, our study relies on pooled data, which limits our ability to draw definitive conclusions about the causal relationship between multimorbidity and workplace productivity. We also recognize that several important factors influencing productivity among patients with multimorbidity, such as the effects of medications, were not accounted for in our study. Fifth, a key limitation is that by restricting the sample to employed individuals or those actively seeking work, our estimates may be biased toward people with multimorbidity who are sufficiently healthy or have well-managed conditions to participate in the labor force. This may underestimate the true productivity losses associated with multimorbidity in the broader population. Sixth, another notable limitation of this study is its reliance on self-reported health conditions and work productivity, which may introduce recall bias. Finally, there are limitations in how key variables were measured. For example, presenteeism was assessed using the SF-36 questionnaire, which was not specifically designed for this purpose and may not fully capture the nuances of presenteeism in the workplace.

## Conclusion

Multimorbidity presents a significant public health challenge in Australia. This study is one of the first to comprehensively investigate the overall productivity losses associated with multimorbidity in the workplace, focusing specifically on absenteeism, presenteeism, and working hour tension. Our findings demonstrate a strong association between multimorbidity and increased rates of absenteeism, presenteeism, and working hour tension, highlighting the substantial indirect economic burden imposed by multiple chronic conditions. These findings carry crucial policy implications, emphasizing the need for workplace policies that support the productivity and well-being of workers living with multimorbidity. To mitigate this burden, it is essential to foster supportive workplace environments that enable all employees, including those living with multiple chronic conditions, to maintain healthy and sustainable employment.

## Supplementary Material

Supplementary_material_FINAL_fdaf132

## Data Availability

This study drew on data from the Household, Income and Labour Dynamics in Australia (HILDA) Survey, conducted by the Melbourne Institute of Applied Economic and Social Research (https://melbourneinstitute.unimelb.edu.au/). Although the HILDA dataset is not openly available, eligible researchers may request access by adhering to the Melbourne Institute's application process and meeting its approval criteria.

## Notes

**Mohammad Afshar Ali,** Postdoctoral Researcher

**Syed Afroz Keramat,** Research Fellow

**Christine Y. Lu,** Professor

## References

[1] Godding R . The persistent challenge of inequality in Australia's health. Med J Aust 2014;201:432. 25332017 10.5694/mja14.c1020

[2] Hiyoshi A, Fukuda Y, Shipley MJ et al. Health inequalities in Japan: the role of material, psychosocial, social relational and behavioural factors. Soc Sci Med 2014;104:201–9. 10.1016/j.socscimed.2013.12.028 24581079

[3] Wang L, Palmer AJ, Cocker F et al. Multimorbidity and health-related quality of life (HRQoL) in a nationally representative population sample: implications of count versus cluster method for defining multimorbidity on HRQoL. Health Qual Life Outcomes 2017;15:7. 10.1186/s12955-016-0580-x 28069026 PMC5223532

[4] ABS . *Microdata: National Health Survey, 2022- AIHW Analysis of Detailed Microdata*. Canberra: Australian Bureau of Statistics, 2023.

[5] Koné Pefoyo AJ, Bronskill SE, Gruneir A et al. The increasing burden and complexity of multimorbidity. BMC Public Health 2015;15:415. 10.1186/s12889-015-1733-2 25903064 PMC4415224

[6] Sum G, Salisbury C, Koh GCH et al. Implications of multimorbidity patterns on health care utilisation and quality of life in middle-income countries: cross-sectional analysis. J Glob Health 2019;9:020413. 10.7189/jogh.09.020413 31448114 PMC6684869

[7] Palladino R, Tayu Lee J, Ashworth M et al. Associations between multimorbidity, healthcare utilisation and health status: evidence from 16 European countries. Age Ageing 2016;45:431–5. 10.1093/ageing/afw044 27013499 PMC4846796

[8] Schofield DJ, Shrestha RN, Passey ME et al. Chronic disease and labour force participation among older Australians. Medical Journal of australia 2008;189:447–50. 10.5694/j.1326-5377.2008.tb02119.x 18928439

[9] Wang L, Si L, Cocker F et al. A systematic review of cost-of-illness studies of multimorbidity. Appl Health Econ Health Policy 2018;16:15–29. 10.1007/s40258-017-0346-6 28856585

[10] Sum G, Ishida M, Koh GCH et al. Implications of multimorbidity on healthcare utilisation and work productivity by socioeconomic groups: cross-sectional analyses of Australia and Japan. PloS One 2020;15:e0232281. 10.1371/journal.pone.0232281 32343739 PMC7188213

[11] Fortin M, Stewart M, Poitras ME et al. A systematic review of prevalence studies on multimorbidity: toward a more uniform methodology. Ann Fam Med 2012;10:142–51. 10.1370/afm.1337 22412006 PMC3315131

[12] Troelstra SA, Straker L, Harris M et al. Multimorbidity is common among young workers and related to increased work absenteeism and presenteeism: results from the population-based Raine study cohort. Scand J Work Environ Health 2020;46:218–27. 10.5271/sjweh.3858 31655849

[13] Cabral GG, Dantas de Souza AC, Barbosa IR et al. Multimorbidity and its impact on workers: a review of longitudinal studies. Saf Health Work 2019;10:393–9. 10.1016/j.shaw.2019.08.004 31890321 PMC6933240

[14] Ubalde-Lopez M, Delclos GL, Benavides FG et al. Measuring multimorbidity in a working population: the effect on incident sickness absence. Int Arch Occup Environ Health 2016;89:667–78. 10.1007/s00420-015-1104-4 26615549 PMC4828479

[15] Meerding WJ, IJzelenberg W, Koopmanschap MA et al. Health problems lead to considerable productivity loss at work among workers with high physical load jobs. J Clin Epidemiol 2005;58:517–23. 10.1016/j.jclinepi.2004.06.016 15845339

[16] Wang PS, Beck A, Berglund P et al. Chronic medical conditions and work performance in the health and work performance questionnaire calibration surveys. J Occup Environ Med 2003;45:1303–11. 10.1097/01.jom.0000100200.90573.df 14665817

[17] Keramat SA, Alam K, Gow J et al. A longitudinal exploration of the relationship between obesity, and long term health condition with presenteeism in Australian workplaces, 2006-2018. PloS One 2020;15:e0238260. 10.1371/journal.pone.0238260 32845941 PMC7449460

[18] Unmuessig V, Fishman PA, Vrijhoef HJM et al. Association of controlled and uncontrolled hypertension with workplace productivity. The Journal of Clinical Hypertension 2016;18:217–22. 10.1111/jch.12648 26279464 PMC8031570

[19] Uribe JM, Pinto DM, Vecino-Ortiz AI et al. Presenteeism, absenteeism, and lost work productivity among depressive patients from five cities of Colombia. Value in health regional issues 2017;14:15–9. 10.1016/j.vhri.2017.03.001 29254536

[20] Ishida M, Hulse ES, Mahar RK et al. The joint effect of physical multimorbidity and mental health conditions among adults in Australia. Prev Chronic Dis 2020;17:E157. 33301391 10.5888/pcd17.200155 PMC7769083

[21] Watson N . Finding your way around the HILDA survey data. Australian Economic Review 2021;54:554–64. 10.1111/1467-8462.12437

[22] Wilkins R . *Families, Incomes, and Jobs. Volume 8: A Statistical Report on Waves 1 to 10 of the Household Income and Labour Dynamics in Australia Survey*. Melbourne Institute of Applied Economic and Social Research Faculty of Business and Economics: Melbourne, 2013.

[23] Keramat SA, Hashmi R, Aregbeshola BS et al. Informal caregiving provision for disabled or elderly in the families and work productivity: evidence from 11 waves of an Australian population-based cohort. Pharmacoeconomics 2023;41:1117–36. 10.1007/s40273-023-01283-6 37338746 PMC10449655

[24] Kler P, Potia AH, Shankar S. Underemployment in Australia: a panel investigation. Applied Economics Letters 2018;25:24–8. 10.1080/13504851.2017.1290770

[25] Keramat SA, Perales F, Alam K et al. Multimorbidity and health-related quality of life amongst indigenous Australians: a longitudinal analysis. Qual Life Res 2024;33:195–206. 10.1007/s11136-023-03500-3 37587324 PMC10784343

[26] Wang L, Cocker F, Kilpatrick M et al. The associations of multimorbidity with health-related productivity loss in a large and diverse public sector setting: a cross-sectional survey. J Occup Environ Med 2018;60:528–35. 10.1097/JOM.0000000000001243 29200192

[27] Bubonya M, Cobb-Clark DA, Wooden M. Mental health and productivity at work: does what you do matter? Labour Economics 2017;46:150–65. 10.1016/j.labeco.2017.05.001

[28] Keramat SA, Comans T, Pearce A et al. Psychological distress and productivity loss: a longitudinal analysis of Australian working adults. Eur J Health Econ 2025, 1–22. 10.1007/s10198-025-01764-9 PMC12572030 40304834

[29] Mohamed R, Patel J, Shaikh NF et al. Absenteeism-related wage loss associated with multimorbidity among employed adults in the United States. J Occup Environ Med 2021;63:508–13. 10.1097/JOM.0000000000002180 34048383

[30] Pearce AM, Hanly P, Timmons A et al. Productivity losses associated with head and neck cancer using the human capital and friction cost approaches. Appl Health Econ Health Policy 2015;13:359–67. 10.1007/s40258-015-0155-8 25691128

[31] Schofield DJ, Callander EJ, Kelly SJ et al. Working beyond the traditional retirement age: the influence of health on Australia’s older workers. J Aging Soc Policy 2017;29:235–44. 10.1080/08959420.2016.1246319 27732170

[32] Fouad AM, Waheed A, Gamal A et al. Effect of chronic diseases on work productivity: a propensity score analysis. J Occup Environ Med 2017;59:480–5. 10.1097/JOM.0000000000000981 28486344

[33] Pourhabib A, Sabzi Z, Yazdi K et al. Facilitators and barriers to return to work in patients after heart surgery. J Educ Health Promot 2022;11:310. 10.4103/jehp.jehp_70_22 36439004 PMC9683457

[34] Tiedtke C, Donceel P, Knops L et al. Supporting return-to-work in the face of legislation: stakeholders' experiences with return-to-work after breast cancer in Belgium. J Occup Rehabil 2012;22:241–51. 10.1007/s10926-011-9342-0 22105670

[35] Hanly P, Pearce A, Sharp L. Cancer and productivity loss in the Irish economy: an employer's perspective. Ir J Managt 2017;36:5–20. 10.1515/ijm-2017-0003

