# Supplementary material for: Workplace productivity losses due to multimorbidity: findings from an Australian longitudinal population survey, 2009–21
Source: J Public Health (Oxf). 2025 Oct 10;48(1):4–18. doi: 10.1093/pubmed/fdaf132 (PMC13017662; doi:10.1093/pubmed/fdaf132)
Supplement: Supplementary_material_FINAL_fdaf132 [file supplementary_material_final_fdaf132.docx]

**Supplementary material /Appendix**

Table A1: Description of the control variables.

| **Variables** | **Measure** |
| --- | --- |
| **Socio-demographic characteristics** | |
| Age (years) | 0 = 15-24  1 = 25-39  2 = 40-64  3 = 65 and above |
| Gender | 0 = Male  1 = Female |
| Relationship status | 0 = Partnered (in a registered marriage or de facto relationship)  1 = Unpartnered (never married, separated but not divorced, divorced, or widowed) |
| Highest level of educational attainment | 0 = Year 12 and below (year 12, and year 11 and below)  1 = Professional qualification (advance diploma or diploma, and certificate III or IV)  2 = University qualification (postgraduate—masters or doctorate, graduate diploma or certificate, bachelor or honours) |
| Household yearly  disposable income | 0 = Quintile 1 (poorest)  1 = Quintile 2 (poorer)  2 = Quintile 3 (middle)  3 = Quintile 4 (richer)  4 = Quintile 5 (richest) |
| Indigenous status | 0 = Not of Indigenous origin  1 = Indigenous origin (Aboriginal, Torres Strait Islander, and both Aboriginal and Torres Strait  Islander) |
| Region of residence | 0 = Major city  1 = Regional or remote area including inner regional, outer regional, remote and very remote Australia |
| **Health-related characteristics** | |
| Weight category | 0 = Underweight (BMI less than 18.50)  1 = Healthy weight (BMI 18.50–24.99)  2 = Overweight or pre-obese (BMI 25.00–29.99)  3 = Obese (BMI 30 or greater) |
| Long-term health condition or disability | 0 = No  1 = Yes |
| **Health-related behaviours** | |
| Smoking status | 0 = Non-smoker (never smoked, and former smoker)  1 = Current smoker (smoke daily, smoke at least weekly and smoke less often than weekly) |
| Alcohol consumption | 0 = Non-drinker (never drunk and ex-drinker)  1 = Current drinker (only rarely, 1–2 days, 2–3 days, 3–4 days, 5–6 days per week and every day) |
| Physical activity | 0 = Less than the recommended level (not at all, less than once, 1–2 and 3 times a week)  1 = Recommended level (> 3 times a week and every day) |
| Hospital admission | 0 = Yes  1 = No |
| Functional disability | 0 = Yes  1 = No |
| **Job-related characteristics** | |
| Firm size | 0 = Small (less than 20 employees)  1 = Medium (20–99 employees)  2 = Large (at least100 employees) |
| Employment contract | 0 = Permanent  1 = Fixed term  2 = Casual |
| Occupation | 0 = Professional  1 = Manager  2 = Technician and trade workers  3 = Community and personal service workers  4 = Clerical and administrative workers  5 = Sales workers  6 = Machinery operators and drivers  7 = Labourers |
| Supervisory responsibilities | 0 = Yes  1 = No |
| Union membership | 0 = Yes  1 = No |
| Paid holiday or sick leave | 0 = Yes  1 = No |
| Overall job satisfaction | 0–10 Likert scale (higher score indicates more job satisfaction) |

Table A2: Distribution of the analytic sample (sociodemographic, health and job-related characteristics) by cohort (excluding unemployed persons).

| **Variable** | **No serious illness**  **(n =24,037)** | | **One**  **serious illness**  **(n =4,475)** | | **Two or more**  **serious illness**  **(n =3,061)** | | **All cohort (n = 31,573)** | |
| --- | --- | --- | --- | --- | --- | --- | --- | --- |
|  | **n** | **%** | **n** | **%** | **n** | **%** | **n** | **%** |
| **Age** |  |  |  |  |  |  |  |  |
| 15-24 years | 4741 | 19.72 | 479 | 10.70 | 385 | 12.58 | 5605 | 17.75 |
| 25-39 years | 8947 | 37.22 | 1222 | 27.31 | 771 | 25.19 | 10940 | 34.65 |
| 40-64 years | 9927 | 41.30 | 2452 | 54.79 | 1584 | 51.75 | 13963 | 44.22 |
| 65 years and above | 422 | 1.76 | 322 | 7.20 | 321 | 10.49 | 1065 | 3.37 |
| **Gender** |  |  |  |  |  |  |  |  |
| Male | 12472 | 51.89 | 2146 | 47.96 | 1248 | 40.77 | 15866 | 50.25 |
| Female | 11565 | 48.11 | 2329 | 52.04 | 1813 | 59.23 | 15707 | 49.75 |
| **Relationship status** |  |  |  |  |  |  |  |  |
| Partnered | 15296 | 63.64 | 2917 | 65.18 | 1783 | 58.25 | 19996 | 63.33 |
| Unpartnered | 8741 | 36.36 | 1558 | 34.82 | 1278 | 41.75 | 11577 | 36.67 |
| **Highest education level** |  |  |  |  |  |  |  |  |
| Year 12 and below | 8139 | 33.86 | 1353 | 30.23 | 1000 | 32.67 | 10492 | 33.23 |
| Professional qualifications | 9402 | 39.11 | 1944 | 43.44 | 1433 | 46.81 | 12779 | 40.47 |
| University qualifications | 6496 | 27.03 | 1178 | 26.32 | 628 | 20.52 | 8302 | 26.29 |
| **Household yearly disposable income** |  |  |  |  |  |  |  |  |
| Q1 (poorest) | 4690 | 19.54 | 796 | 17.82 | 775 | 25.38 | 6261 | 19.86 |
| Q2 | 4917 | 20.49 | 791 | 17.71 | 605 | 19.82 | 6313 | 20.03 |
| Q3 | 4957 | 20.66 | 815 | 18.24 | 544 | 17.82 | 6316 | 20.04 |
| Q4 | 4774 | 19.89 | 987 | 22.10 | 554 | 18.15 | 6315 | 20.04 |
| Q5 (richest) | 4660 | 19.42 | 1078 | 24.13 | 575 | 18.83 | 6313 | 20.03 |
| **Indigenous status** |  |  |  |  |  |  |  |  |
| Not of Indigenous origin | 24011 | 99.89 | 4469 | 99.87 | 3057 | 99.87 | 31537 | 99.89 |
| Indigenous origin | 26 | 0.11 | 6 | 0.13 | 4 | 0.13 | 36 | 0.11 |
| **Region of residence** |  |  |  |  |  |  |  |  |
| Major city | 16988 | 70.67 | 3064 | 68.47 | 2062 | 67.36 | 22114 | 70.04 |
| Regional city and remote area | 7049 | 29.33 | 1411 | 31.53 | 999 | 32.64 | 9459 | 29.96 |
| **BMI** |  |  |  |  |  |  |  |  |
| Underweight | 557 | 2.32 | 62 | 1.39 | 42 | 1.37 | 661 | 2.09 |
| Healthy weight | 10529 | 43.80 | 1432 | 32.00 | 786 | 25.68 | 12747 | 40.37 |
| Overweight | 8424 | 35.05 | 1642 | 36.69 | 973 | 31.79 | 11039 | 34.96 |
| Obesity | 4527 | 18.83 | 1339 | 29.92 | 1260 | 41.16 | 7126 | 22.57 |
| **Long-term condition or disability** |  |  |  |  |  |  |  |  |
| Yes | 19986 | 83.15 | 3822 | 85.41 | 2405 | 78.57 | 26213 | 83.02 |
| No | 4051 | 16.85 | 653 | 14.59 | 656 | 21.43 | 5360 | 16.98 |
| **Smoking status** |  |  |  |  |  |  |  |  |
| Non-smoker | 19986 | 83.15 | 3822 | 85.41 | 2405 | 78.57 | 26213 | 83.02 |
| Current smoker | 4051 | 16.85 | 653 | 14.59 | 656 | 21.43 | 5360 | 16.98 |
| **Alcohol consumption** |  |  |  |  |  |  |  |  |
| Current drinker | 3198 | 13.30 | 598 | 13.36 | 474 | 15.49 | 4270 | 13.52 |
| Non-drinker | 20839 | 86.70 | 3877 | 86.64 | 2587 | 84.51 | 27303 | 86.48 |
| **Physical activity** |  |  |  |  |  |  |  |  |
| Less than the recommended level | 15016 | 62.47 | 2925 | 65.36 | 2232 | 72.92 | 20173 | 63.89 |
| Recommended level | 9021 | 37.53 | 1550 | 34.64 | 829 | 27.08 | 11400 | 36.11 |
| **Firm size** |  |  |  |  |  |  |  |  |
| Small (1-19 employees) | 10240 | 42.60 | 1874 | 41.88 | 1425 | 46.55 | 13539 | 42.88 |
| Medium (20-99 employees) | 6659 | 27.70 | 1226 | 27.40 | 776 | 25.35 | 8661 | 27.43 |
| Large (>= 100 employees) | 7138 | 29.70 | 1375 | 30.73 | 860 | 28.10 | 9373 | 29.69 |
| **Employment contract** |  |  |  |  |  |  |  |  |
| Casual | 6128 | 21.89 | 1006 | 20.46 | 892 | 25.25 | 3478 | 9.54 |
| Fixed term | 2726 | 9.74 | 441 | 8.97 | 311 | 8.81 | 24941 | 68.43 |
| Permanent | 19143 | 68.38 | 3469 | 70.57 | 2329 | 65.94 | 8026 | 22.02 |
| **Employment nature** |  |  |  |  |  |  |  |  |
| Fulltime | 16273 | 67.70 | 2937 | 65.63 | 1729 | 56.48 | 20939 | 66.32 |
| Parttime | 7764 | 32.30 | 1538 | 34.37 | 1332 | 43.52 | 10634 | 33.68 |
| Unemployed | N/A | N/A | N/A | N/A | N/A | N/A | N/A | N/A |
| **Occupation** |  |  |  |  |  |  |  |  |
| Managers | 3286 | 13.67 | 678 | 15.15 | 378 | 12.35 | 4342 | 13.75 |
| Professionals | 6304 | 26.23 | 1176 | 26.28 | 697 | 22.77 | 8177 | 25.90 |
| Technicians and trades workers | 3192 | 13.28 | 545 | 12.18 | 315 | 10.29 | 4052 | 12.83 |
| Community and personal service workers | 2683 | 11.16 | 527 | 11.78 | 468 | 15.29 | 3678 | 11.65 |
| Clerical and administrative workers | 3181 | 13.23 | 607 | 13.56 | 430 | 14.05 | 4218 | 13.36 |
| Sales workers | 2122 | 8.83 | 317 | 7.08 | 263 | 8.59 | 2702 | 8.56 |
| Machinery operators and drivers | 1311 | 5.45 | 284 | 6.35 | 206 | 6.73 | 1801 | 5.70 |
| Labourers | 1958 | 8.15 | 341 | 7.62 | 304 | 9.93 | 2603 | 8.24 |
| **Supervisory responsibilities** |  |  |  |  |  |  |  |  |
| No | 13317 | 55.40 | 2574 | 57.52 | 1908 | 62.33 | 17799 | 56.37 |
| Yes | 10720 | 44.60 | 1901 | 42.48 | 1153 | 37.67 | 13774 | 43.63 |
| **Union membership** |  |  |  |  |  |  |  |  |
| No | 18866 | 78.49 | 3451 | 77.12 | 2314 | 75.60 | 24631 | 78.01 |
| Yes | 5171 | 21.51 | 1024 | 22.88 | 747 | 24.40 | 6942 | 21.99 |
| **Paid holiday or sick leave** |  |  |  |  |  |  |  |  |
| No | 5740 | 23.88 | 993 | 22.19 | 810 | 26.46 | 7543 | 23.89 |
| Yes | 18297 | 76.12 | 3482 | 77.81 | 2251 | 73.54 | 24030 | 76.11 |
| **Hospital admission** |  |  |  |  |  |  |  |  |
| No | 22413 | 93.24 | 4031 | 90.08 | 2533 | 82.75 | 28977 | 91.78 |
| Yes | 1624 | 6.76 | 444 | 9.92 | 528 | 17.25 | 2596 | 8.22 |
| **Functional disability** |  |  |  |  |  |  |  |  |
| No | 22984 | 95.62 | 3910 | 87.37 | 2137 | 69.81 | 29031 | 91.95 |
| Yes | 1053 | 4.38 | 565 | 12.63 | 924 | 30.19 | 2542 | 8.05 |
| **Overall job satisfaction, mean (SD)** | 7.74 (1.51) | | 7.78 (1.56) | | 7.69 (1.71) | | 7.74 (1.54) | |

Table A3: Additional absent days of a person with two or more conditions compared to a person with no serious illness.

| Productivity measures | Person with two or more conditions |
| --- | --- |
| Additional absent days by two measures |  |
| Mean comparison, unadjusted | 0.90 |
| Negative binomial regression marginal effects, adjusted | 1.20 |

Table A4: Weekly and daily gross wages and salary.

| Gross daily wages and salary (all) | Sources | |
| --- | --- | --- |
|  | HILDA sample | ABS statistics |
| All | 221.00 | 275.32 |
| Aged 15-24 years | 114.00 | N/A |
| Aged 25-39 years | 248.00 | N/A |
| Aged 40-64 years | 255.00 | N/A |
| Aged 65 years and above | 145.00 | N/A |

Table A5: Rates of multimorbidity in the study cohort.

| **Serious illness** | **Age specific rate** | | | | **Overall rate** |
| --- | --- | --- | --- | --- | --- |
|  | 15-24 | 25-39 | 40-64 | 65 and above | All age group (%) |
|  | n (%) | n (%) | n (%) | n (%) | N (%) |
| Any type of cancer | 0 (0.00) | 17 (0.15) | 148 (1.16) | 52 (6.52) | 217 (0.69) |
| Anxiety | 473 (7.28) | 753 (6.58) | 668 (5.22) | 38 (4.77) | 1932 (6.13) |
| Arthritis or osteoporosis | 14 (0.22) | 122 (1.07) | 756 (5.91) | 194 (24.34) | 1086 (3.44) |
| Asthma | 154 (2.37) | 341 (2.98) | 507 (3.97) | 51 (6.40) | 1053 (3.34) |
| Chronic bronchitis or emphysema | 5 (0.08) | 14 (0.12) | 78 (0.61) | 23 (2.89) | 120 (0.38) |
| Type 1 diabetes | 2 (0.03) | 16 (0.14) | 39 (0.31) | 9 (1.13) | 66 (0.21) |
| Type 2 diabetes | 3 (0.05) | 21 (0.18) | 311 (2.43) | 94 (11.79) | 429 (1.36) |
| Depression | 427 (6.57) | 683 (5.96) | 723 (5.66) | 44 (5.52) | 1877 (5.95) |
| High blood pressure or hypertension | 16 (0.25) | 106 (0.93) | 868 (6.79) | 240 (30.11) | 1230 (3.90) |
| Heart disease | 3 (0.05) | 13 (0.11) | 196 (1.53) | 85 (10.66) | 297 (0.94) |
| Any other serious circulatory condition (eg stroke, hardening of the arteries) | 4 (0.06) | 22 (0.19) | 145 (1.13) | 44 (5.52) | 215 (0.68) |
| Other mental illness | 143 (2.20) | 166 (1.45) | 101 (0.79) | 2 (0.25) | 412 (1.31) |

Table A6: Effects of severity of conditions and functional disability on hospital admission.

| **Variables** | **Outcome: Hospital admission**  **Adjusted OR****  **(95%CI)** |
| --- | --- |
| **Chronic illness** |  |
| No serious illness (ref) |  |
| One serious illness | 1.20*  [1.06 – 1.36] |
| At least two serious illness | 2.04*  [1.79 – 2.33] |
| **Interaction effect**  **[multimorbidity × functional disability]** |  |
| No |  |
| Yes | 0.85  [0.67 – 1.10] |

Note: 1) 95% confidence intervals are reported in parentheses; 2) * Indicate significance at the 5% level; 3) Abbreviation: AOR, adjusted odds ratio; Ref, reference category; 4)** denotes regression estimates are adjusted for covariates included in the main models (Table 3).

Table A7: Effects of severity of conditions and functional disability on outcome variables.

| **Variables** | **Model 1: Absenteeism**  **Adjusted IRR****  **(95%CI)** | **Model 2: Presenteeism**  **Adjusted OR****  **(95%CI)** | **Model 3: Working hour tension**  **Adjusted IRR****  **(95%CI)** |
| --- | --- | --- | --- |
| **Chronic illness** |  |  |  |
| No serious illness (ref) |  |  |  |
| One serious illness | 1.05*  [1.00 – 1.09] | 1.08*  [1.07– 1.10] | 0.94*  [0.81 – 1.10] |
| At least two serious illness | 0.99*  [0.94 – 1.07] | 1.07*  [1.05– 1.08] | 1.32*  [1.07 – 1.61] |
| **Hospital admission** |  |  |  |
| No |  |  |  |
| Yes | 1.77*  [1.68 – 1.87] | 1.07*  [1.05– 1.08] | 1.26*  [1.05 – 1.51] |
| **Interaction effect**  **[multimorbidity × functional disability]** |  |  |  |
| No |  |  |  |
| Yes | 1.08  [0.96 – 1.23] | 0.96  [0.92 – 1.01] | 0.93  [0.61 – 1.44] |

Note: 1) 95% confidence intervals are reported in parentheses; 2) * Indicate significance at the 5% level; 3) Abbreviation: IRR, incidence rate ratio; AOR, adjusted odds ratio; Ref, reference category; 4)** denotes regression estimates are adjusted for covariates included in the main models (Table

| 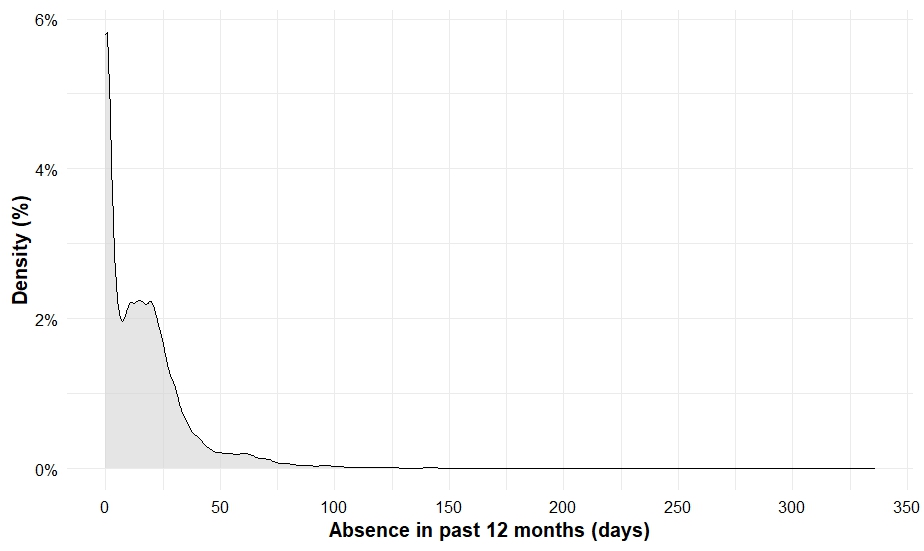 |
| --- |
| 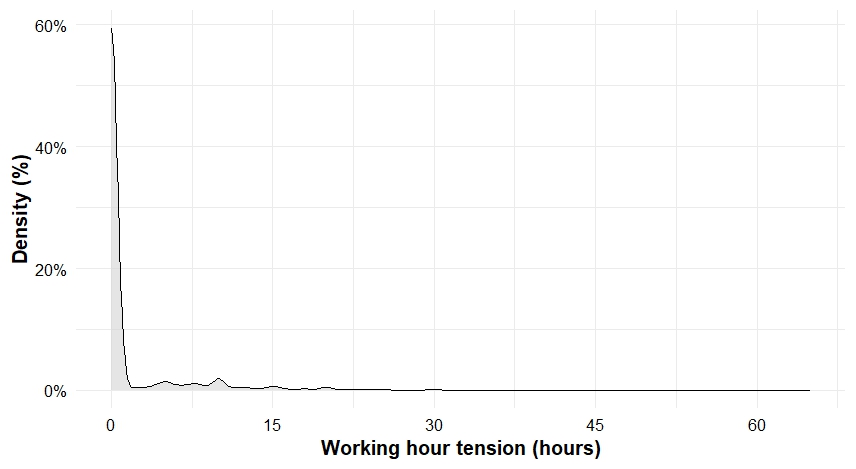 |

Figure A1: Distribution of number of days absent and work hours lost in the past 12 months.
